# Editorial: Innovative food technologies for a sustainable future: bridging science, processing, and circularity

**DOI:** 10.3389/fnut.2026.1964683

**Published:** 2026-09-14

**Authors:** Margherita Modesti

**Affiliations:** Department for Innovation of Biological, Agrofood and Forest Systems (DIBAF), University of Tuscia, Viterbo, Italy

**Keywords:** circularity, digitalization, food processing, innovative food technologies, sustainable food systems

The transition toward more sustainable food systems requires more than the development of individual technological solutions. It calls for an integrated rethinking of how foods are produced, processed, preserved, monitored, and ultimately accepted by consumers. In this context, innovation can contribute to sustainability when technological advances are connected with resource efficiency, circularity, nutritional quality, product stability, and consumer needs. The contributions collected in this Research Topic illustrate this multidimensional perspective, spanning fermentation and alternative raw materials, biopolymer-based solutions, digital technologies for food quality management, and consumer acceptance of emerging processing technologies (Figure 1).

**Figure 1:**
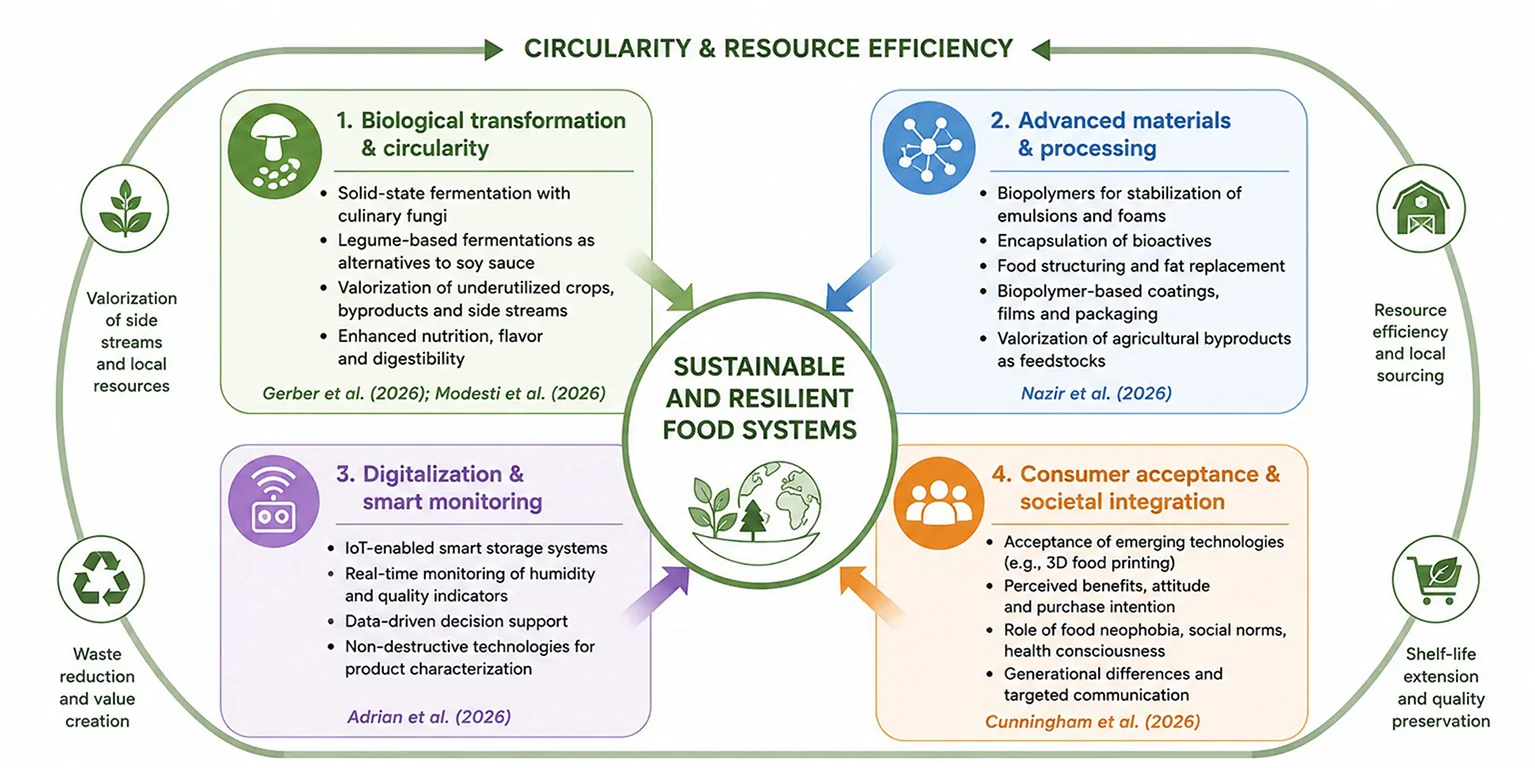
Infographic of the key innovation pathways toward sustainable and resilient food systems.

A recurring theme across the Research Topic is the potential to obtain greater value from biological resources through processing strategies that combine innovation with circularity. Gerber et al. examine solid-state fermentation with culinary fungi as a platform for transforming cereals, legumes, oilseed meals, and agricultural side streams into nutrient-dense and minimally processed foods. The evidence indicates that SSF can improve protein quality and nutrient bioavailability, reduce antinutritional factors, and generate desirable flavor and texture characteristics. Importantly, this approach connects nutritional improvement with the valorization of underutilized resources and potentially shorter production chains. Nevertheless, its transition toward large-scale implementation requires better process standardization, control of substrate and process variability, integrated sensory and safety assessment, and robust techno-economic and life-cycle evaluations.

The potential of fermentation to connect traditional processing knowledge with contemporary sustainability goals is also demonstrated by Modesti et al. Their comparison of conventional soy sauces with fermented sauces produced from locally sourced peas, chickpeas, and lentils shows that alternative substrates can generate products characterized by substantial chemical and sensory complexity and strong consumer acceptance. Electronic nose and near-infrared spectroscopy further demonstrated the potential of rapid, non-destructive approaches for product discrimination. Particularly relevant from a circular perspective is the use of locally sourced legumes, including agricultural materials that would otherwise be discarded, demonstrating how traditional fermentation can be repositioned within a waste-to-value strategy. At the same time, the study highlights the importance of considering processing conditions, formulation, and production practices alongside raw-material effects when evaluating alternative products.

Material innovation represents another important component of this transition. Nazir et al. provide an integrated overview of biopolymers as multifunctional tools for emulsion stabilization, encapsulation, food structuring, fat replacement, and packaging. Their contribution demonstrates how renewable materials can simultaneously address technological functionality and sustainability, including through the valorization of agri-food byproducts. However, the environmental promise of biopolymers cannot be considered independently of technological and industrial feasibility. Variability in upcycled feedstocks, barrier performance, mechanical properties, regulatory requirements, scalability, and economic feasibility remain important constraints. Future developments therefore need to connect structure–property relationships with realistic applications, techno-economic assessment, and life-cycle analysis.

Digitalization provides a complementary pathway by allowing food quality to be monitored rather than assessed only retrospectively. Adrian et al. developed an Internet of Things (IoT)-based humidity-responsive container for rambak crackers after identifying relative-humidity thresholds associated with sensory deterioration. The system illustrates how physicochemical knowledge of food deterioration can be translated into real-time monitoring and decision support, with potential benefits for quality management and reduction of food and economic losses. Importantly, the prototype also illustrates the challenges involved in translating proof-of-concept digital technologies to realistic conditions: sensor responsiveness, system configuration, container design, and validation across foods with different hygroscopic properties require further investigation.

Yet technological feasibility alone does not ensure adoption. Cunningham et al. address this dimension through their investigation of consumer intention toward 3D-printed food. Awareness, perceived benefits, and attitude emerged as important drivers across Generation X and Generation Y consumers, while food neophobia, social norms, and health consciousness showed generation-dependent effects. These findings emphasize that emerging food technologies operate within social and cultural contexts and that communication and implementation strategies cannot assume homogeneous consumer responses. Future studies incorporating real consumption behavior, sustainability perceptions, ethical considerations, and AI-enabled personalization could further connect technological capabilities with societal expectations.

Taken together, the contributions to this Research Topic suggest that the future of sustainable food technology lies not in a single disruptive technology, but in the convergence of complementary approaches. Biological transformations can valorize local resources and side streams; advanced materials can improve functionality and reduce dependence on conventional materials; sensing and digitalization can enable more responsive quality management; and emerging processing technologies can create new forms of production and personalization. Across these domains, however, similar challenges recur: scalability, process standardization, economic feasibility, safety and regulation, environmental assessment, and consumer acceptance.

Bridging science, processing, and circularity therefore requires moving beyond proof-of-concept innovation toward integrated evaluation under realistic food-system conditions. Technologies should ultimately be assessed not only by what they can technically achieve, but also by their capacity to preserve or improve food quality, use resources more efficiently, reduce losses and waste, create value from underutilized materials, and deliver products that consumers are willing to adopt. The studies gathered in this Research Topic provide complementary examples of this transition and highlight the interdisciplinary effort required to translate food innovation into resilient and sustainable food systems.

